# Blood and Saliva Composition in Cancer: Similarities or Differences?

**DOI:** 10.3390/cimb48030308

**Published:** 2026-03-12

**Authors:** Elena I. Dyachenko, Lyudmila V. Bel’skaya

**Affiliations:** Biochemistry Research Laboratory, Omsk State Pedagogical University, 644099 Omsk, Russia; a.dyachenko@omgpu.ru

**Keywords:** saliva, plasma, cancer, correlation, biochemistry, cytokines, tumor markers, growth factors, hormones

## Abstract

Saliva is of great interest for diagnosing and monitoring various diseases, including cancer. Saliva contains a wide range of proteins, some of which are found in blood plasma, while others are unique. Despite the obvious advantages of using saliva for diagnostics and patient monitoring, difficulties in interpreting salivary values remain. Furthermore, the extent to which salivary results correlate with blood test results remains unclear. In this review, we have collected and analyzed all currently available parallel studies of saliva and blood on the nature of changes in biochemical parameters, cytokines, growth factors, hormones, and tumor markers in cancer patients. The most contradictory and divergent changes in saliva and blood were observed when measuring biochemical parameters. Cytokines, growth factors, hormones, and tumor markers in both saliva and blood have a higher reproducibility between independent studies. It is important to consider that the causes and mechanisms behind a particular indicator in saliva may differ from those underlying the same indicators in the blood. Furthermore, these indicators may not always be directly related to cancer. We suggest that comparing identical parameters in blood and saliva is useful in the context of “pathological process routing.” The paper also provides interpretations and hypotheses regarding the causes and nature of changes in saliva and blood composition in cancer.

## 1. Introduction

Interest in saliva for diagnosing various diseases, including cancer, has increased significantly over the past few years [1,2,3]. Saliva is an attractive biological fluid for searching for disease biomarkers, for diagnostic purposes, and for monitoring patient condition [4]. Based on numerous experimental data from a wide range of laboratories, a catalog of 2290 proteins detected in whole saliva was compiled, which was then compared with 2698 blood plasma proteins. Molecular functions, biological processes, and cellular components show significant similarity between the 27% of proteins that are the same in blood and saliva. In addition, approximately 40% of proteins proposed as potential markers of cardiovascular disease, stroke, and cancer can also be detected in whole saliva [5]. Saliva is characterized by ease of collection, storage, and transportation [6]. However, difficulties and questions often arise in interpreting the values of measured parameters in saliva. There is no clear answer to the question of the advisability of conducting parallel studies of saliva and blood composition. Should saliva be considered an independent fluid with its own criteria for normal and abnormal results?

In our view, comparing identical parameters in blood and saliva is useful in the context of “pathological process routing.” Such a comparison is necessary for a better understanding of how the immediate source of a pathological process, such as a tumor, spreads, increases oxidative stress, and activates and reprograms the immune system, leading to damage to tissues, organs, and barriers. As a result, we can observe changes in parameters of saliva and other biological fluids.

However, comparing changes in the same parameters in saliva and plasma and expecting identical changes is a mistake, as these are completely different biological environments. Consequently, the mechanisms by which certain parameters appear in saliva and plasma will differ. Furthermore, saliva and plasma should not be viewed as completely isolated environments, as the barrier separating them becomes less hermetic as the pathological process becomes more aggressive. This means that saliva reflects the changes occurring in the body.

The purpose of this study is to collect existing parallel studies of plasma and saliva parameters. Based on this information, we will hypothesize the causes and mechanisms of the appearance of certain components in saliva that were detected in plasma. We will also determine in which cases changes in saliva parameters are directly related to the oncological process, and in which cases they are indirectly related, through damage to the blood-saliva barrier, inflammatory processes, etc.

## 2. Materials and Methods

A review was conducted up to 31 December 2025, using the databases PubMed, Scopus and Web of Science. The search sentence used was TITLE-ABS-KEY ((cancer OR carcinoma OR neoplasm OR tumour OR tumor OR oncology) AND saliva AND (plasma OR serum OR blood)). Records were screened by the title, abstract, and full text by two independent investigators. At the initial stage, duplicate articles in different databases were deleted; reviews and conference abstracts were excluded from consideration.

The search returned a total of 179 reports from databases after deleting duplicates. We then excluded studies that were not related to the study question or were reviews, conference papers, book chapters, notes, letters or editorials. The final list contained 31 papers.

## 3. Parallel Studies of Biochemical Parameters in Saliva and Plasma in Cancer

Relatively few studies have been conducted in the area of parallel changes in biochemical parameters in saliva and blood in the same patients with cancer (Table 1).

Atrooz et al. analyzed the changes in serum and saliva of such biochemical parameters as total protein, albumin, calcium, alkaline phosphatase (ALP), alanine aminotransferase (ALT), aspartate aminotransferase (AST), lactate dehydrogenase (LDH), gamma-glutamyl transferase (GGT), urea and uric acid in patients with breast cancer [7]. They concluded that serum LDH was statistically significantly different in the breast cancer group compared to the control group. Furthermore, salivary AST and urea were statistically significantly different between breast cancer patients and healthy controls. It was also noted that higher BMI and older age were more significant predictors of breast cancer risk than smoking and breastfeeding history [7].

We noted that biochemical parameters are divided into three groups based on the direction of change: unidirectional changes upward, unidirectional changes downward, and changes in different directions. Thus, ALP, AST, GGT, urea, and uric acid change in the same direction upward in patients with breast cancer both in saliva and serum. ALT decreases equally in patients with breast cancer both in serum and saliva. Total protein, albumin, LDH, and calcium change in different directions. In saliva, total protein and albumin increase, while calcium and LDH increase in serum. Another study compared changes in total protein levels in saliva and serum between groups of patients with breast cancer and healthy controls. The work noted that in patients with breast cancer, serum total protein increases, while in saliva it decreases significantly [8].

Luo et al. demonstrated that adenosyl deaminase can be altered in inflammation and cancer. This enzyme is a purine metabolism enzyme that converts adenosine to inosine [11]. In the human body, ADA exists in two types: ADA1 (intracellular) and ADA2 (secreted). The main function of ADA1 is to break down adenosine, which is toxic to immune cells. However, the mechanisms by which ADA1 levels increase remain poorly understood [20]. Presumably, elevated ADA1 levels in biological fluids may be associated with the release of the enzyme from dying cells at sites of inflammation during tumor growth. ADA2 is a secreted protein expressed by macrophages and dendritic cells [21]. Mutations in both ADA1 and ADA2 can lead to immune system failure [22,23]. A number of studies have reported conflicting data on changes in ADA enzyme activity in saliva in patients with oral cancer [24,25]. Luo et al. showed increased ADA2 activity in saliva in patients with head and neck cancer. The scientists also noted that ADA2 enzyme activity is determined by the type of cancer. Its activity was higher in laryngeal and tonsil cancer than in epiglottic cancer. In addition, the scientists showed that high ADA2 activity in saliva may indicate persistent activation of the immune system in response to the oncological process [11].

Magnesium concentrations were low in both plasma and saliva of patients with oral squamous cell carcinoma compared with those of patients with potentially malignant diseases and healthy individuals [12]. Thus, the concentration of magnesium ions in plasma and saliva can be considered a tumor marker that plays an important role in carcinogenesis.

Farahani et al. showed that the concentration of urea, creatinine, brain-derived creatine kinase (CK-BB), and zinc in both serum and saliva was significantly higher in patients with prostate cancer compared to a group of patients with benign prostatic hyperplasia. In both groups, the concentration of all markers in saliva was lower compared to their concentrations in serum. The scientists noted a positive correlation between the concentrations of urea, creatinine, CK-BB, and zinc in serum and saliva [19].

In another study, scientists conducted a parallel analysis of changes in phosphate levels in the serum and saliva of patients with gastric and colon cancer [10]. A distinctive feature was that phosphates in both serum and saliva were lower in the group of patients with cancer than in the control group. However, phosphate levels were higher in saliva than in serum. As has been mentioned several times, saliva components originate either from the blood or from the cells of the salivary glands. A number of authors conclude that salivary secretion, like urine, is one of the ways to remove waste products [26,27]. There are three key factors that can lead to a decrease in phosphate levels: insufficient food intake, digestive problems, or impaired renal excretion, which can be observed in patients with gastrointestinal cancer. Phosphate, like calcium, is regulated by vitamin D3 and parathyroid hormone (PTH) and is excreted primarily by the kidneys [28]. When examining phosphate levels, the influence of hormones on their content should also be considered. For example, testosterone can decrease phosphate levels [29]. It has been shown that PTH levels increase, while vitamin D3 levels decrease, in gastrointestinal cancer [30,31]. Since increased PTH levels and vitamin D deficiency lead to decreased intestinal phosphate absorption and increased renal excretion, this may be the reason for decreased serum phosphate levels in gastric and colon cancer [32].

One of the most commonly used enzymes for parallel analysis in saliva and blood is LDH in head and neck cancer. A study was conducted in which saliva and blood were collected from healthy controls, patients with oral squamous cell carcinoma (OSCC), oral lichen planus (OLP), and oral lichenoid reactions (OLRs). Thus, in patients with oral squamous cell carcinoma, LDH activity in serum and saliva was significantly higher than in the groups with OLRs, OLP, and healthy controls. A significant correlation was also noted between LDH activity in serum and saliva [13,14]. As is known, LDH release occurs during cell membrane destruction, which allows us to estimate the rate of cell death in various pathological conditions [33]. Cancer cells, during their own death, as well as healthy tissue adjacent to the tumor, are damaged by cancer cells, which leads to the release of LDH into the bloodstream [34,35]. This has been shown in many types of cancer, such as colorectal cancer, breast cancer and cervical cancer [36,37,38]. It is also reported that LDH in the blood serum also increases in oral cancer [35,39]. The activity of the LDH enzyme is due to an increase in the mitotic index, with subsequent formation of lactic acid by cancer cells. Thus, more pronounced dysplastic or malignant changes will further increase the LDH level [40]. The authors conclude that variable LDH levels in normal, potentially malignant, and malignant groups may be associated with different degrees of dysplasia and histological changes [14]. Additionally, a number of independent studies by groups of scientists have concluded that salivary LDH is preferable to serum LDH for the early diagnosis and monitoring of malignant neoplasms and potentially malignant neoplasms of the oral cavity [41,42].

Using glioblastoma as an example, Muller Bark et al. showed that the metabolomic and lipidomic composition of blood and saliva was more diverse and altered in malignant lesions compared to the control group [17]. The scientists noted an increased formation of adenosine-5′-monophosphate, 3-hydroxykynurenine, dihydroorotate, uridine-5′-diphosphate and cis-aconitate in patients with an unfavorable outcome, compared to patients with a favorable outcome, both before and after surgery. These metabolites affect the pentose phosphate metabolism pathways and the Warburg effect. Regarding the lipid profile, patients with an unfavorable outcome showed higher heterogeneity in lipid amounts and fewer associations between markers compared to the group with a favorable outcome [43]. The scientists also showed that kynurenine and creatine, as well as glycolytic metabolite levels, were higher in plasma than in saliva in patients with glioblastoma. Changes in tyrosine, phenylalanine, glucose, creatine, and creatinine levels showed a significant correlation with tumor grade. Meanwhile, citrate, succinate, tricarboxylic acid cycles, and oxamic acid levels were reduced in plasma. In saliva, scientists have noted a strong increase in the amount of ceramides in saliva compared to blood plasma. Hwang et al. showed the opposite results for the concentration of ceramides (B18:1/24:0) in saliva and blood, namely, their decrease in patients with non-small cell lung cancer (NSCLC) compared to healthy controls [18]. At the same time, the total amount of triglycerides (TG) in both plasma and blood was increased in patients with NSCLC compared to healthy controls. Scientists noted an increase in triglycerides such as 50:2, 52:5, 54:6 both in blood plasma and saliva in NSCLC. Phosphatidylinositol 38:4 was also decreased in saliva and blood plasma. Phosphatidylserine 36:1 was detected only in saliva and its amount decreased as the disease progressed. In conclusion, the scientists noted that they identified 27 lipids in saliva and 10 lipids in blood plasma that could be used as potential biomarkers for NSCLC. Lipids are known to be key components of cell membranes and can also be present in various biological fluids of the body. Lipids are involved in signal transduction, cell proliferation and death, and energy storage [44,45]. Metabolic reprogramming of cells is known to be a hallmark of cancer cells. Thus, they are capable of obtaining a continuous source of energy and structural elements for unlimited growth [17]. These metabolites can be measured in blood, saliva, and other biological fluids. Saliva is the most biologically relevant material due to its noninvasive collection approach for monitoring metabolic changes in the human body during oncology [46,47].

Matrix metalloproteinases (MMPs) are another important and frequently used indicator in the diagnosis of oncological diseases. MMPs are proteases that are involved in tissue modeling, stem cell proliferation and differentiation, and cell apoptosis, as well as inflammation, tissue degeneration, and cancer. They can cleave and/or activate proteins that can initiate, participate in tumor progression, and metastasis. This allows MMPs to be considered as potential diagnostic, prognostic, and therapeutic targets for cancer patients [48,49]. Zohreh Dalirsani et al. conducted a parallel study of MMP-2 and MMP-9 in serum and saliva of patients with head and neck squamous cell carcinoma (HNSCC). Briefly, MMP-2 and MMP-9 are proteases that play a role in the removal of extracellular matrix (ECM) by cleaving collagen IV and, thus, are involved in tumor invasion and metastasis. The researchers showed that patients with head and neck squamous cell carcinoma had higher MMP-9 levels in serum and saliva than controls. A direct correlation was also shown between salivary and serum MMP-9 in patients with cancer, but not in healthy controls. However, no significant difference was found in MMP-2 levels between healthy controls and the HNSCC group [15].

In contrast, another study of patients with laryngeal and pharyngeal cancer showed no statistically significant differences in MMP-9 levels between the study and control groups, in either serum or saliva. However, the concentration of MMP-3 in both serum and saliva in cancer patients was statistically significantly different from that in the control group [16].

Rezaei et al. conducted a meta-analysis to evaluate the differences in serum and salivary MMP levels between patients with oral squamous cell carcinoma (OSCC) and healthy controls. The results of the study showed that serum MMP-7 and MPP-9 levels, as well as salivary MMP-1 and MPP-9 levels, were significantly higher in patients with OSCC than in the control group [50].

In order to understand the mechanisms of MMP entry into saliva, it is necessary to devote a few words to the causes and mechanisms of MMP activation. There is still no precise understanding of which molecules activate a particular MMP, but the general principles that serve their synthesis and transition to an active state have already been described by a number of scientists. Thus, the activation and synthesis of MMP can result from oxidative damage to the interaction of the thiol of the conserved cysteine residue of the prodomain with the zinc ion of the catalytic center, modification of the extracellular matrix [51], allosteric disruption of the zymogen, reduction in the free thiol by oxidants or non-physiological reagents, such as alkylating agents, heavy metal ions and disulfides [52]. Oncological processes are inextricably linked with inflammation, attracting various cells of the immune response, including neutrophils. Neutrophils secrete MMP directly in vesicles, superoxide oxygen and MPO (myeloperoxidase) [53]. Oxygen superoxide undergoes dismutation to form hydrogen peroxide [54]. Positively charged MPO readily interacts with negatively charged ECM proteins and attracts hydrogen peroxide to form hypochlorous acid HOCl [55,56,57]. This leads to modification of ECM proteins, which induces the processes of MMP synthesis and activation [58]. Thus, MMP activation can occur directly through oxidation or indirectly, through modification of ECM proteins. Allosteric disruption of zymogen occurs through the action of furin or, in the case of MMP-2, through the action of pericellular MMP-14 [52]. Oncological processes are accompanied by an increase in the content of free heavy metals, which also leads to disruption of the thiol prodomain of MMP and activation of zymogen [59,60].

There are separate publications describing additional mechanisms of MMP activation. Thus, MMP3 activation can occur due to the action of inflammatory mediators, growth factors or mechanical stress. It is known that β-Estradiol (a hormone capable of modulating MMP-3 expression), calcium (a second messenger activates calmodulin-dependent kinases and phosphorylates transcription factors that affect MMP-3 levels), human angiotensin II (a GPCR agonist activates MAPK signaling pathways and increases the level of MMP-3 mRNA through AP-1) and PGE2 (binds to estrogen receptors, increasing the level of cAMP and activating the PKA pathway, which leads to the activation of MMP-3 by CREB), cathepsin G and neutrophil elastase also contribute to the activation of MMP-3. Activation of MMP-9 occurs due to the action of MMP-3, plasmin and fibrinonectin, furin [61]. In addition, MMP9 activation can occur in two ways: oxidative (via Cys oxidation) and via a modified extracellular matrix with the formation of hypochlorous acid HOCL, which interacts with ECM proteins to form chloramines. Chloramines can be formed on HSA, undergo modification, and affect MCH II and activate pro-MMP-9 [62]. Activation of MMP-2 (also due to MMP-3) and MMP-7 occurs via a mechanism involving the oxidation of the conserved propeptide Cys (the “cysteine switch”) [63,64,65].

We hypothesize three hypothetical scenarios for the entry of MMPs into saliva. The first scenario is that cancer cells and their microenvironment produce large amounts of ROS, attracting immune cells and pro-inflammatory cytokines [66], which induce synthesis and activate zymogen. Subsequently, active forms of MMPs enter saliva through the blood-salivary barrier (BSB) via the blood and lymph flow. The second scenario is that ROS, immune cells, and pro-inflammatory cytokines reach the BSB via the blood flow, where they damage the ECM. This results in direct oxidative damage to BSB ECM proteins and modification of ECM proteins, with activation and release of MMPs into saliva. The third scenario is that neutrophils and other immune cells enter saliva through the intact BSB, where both the release and activation of MMPs occur from neutrophils. Thus, the presence of MMP in saliva can be realized through one of the above-proposed options, or through all three.

It is worth mentioning that the discrepancy between interlaboratory results for MMPs in the blood may be due to the procedure for collecting the biomaterial and the blood coagulation cascade, which can significantly affect the concentration of both proteins and metabolites in plasma and serum. This may be the cause of possible “preanalytical errors” associated with both the collection and processing of blood. Thus, it was noted that the amount of MMP-1, -2, -7, -8 and -9 in blood serum was significantly higher than the amount of the same MMPs in citrated plasma. This observation serves as a good indicator that blood serum may not always adequately and accurately reflect the content of certain biochemical parameters in the blood. In this regard, the search for new biomaterials and methods for their collection is a relevant area of research [67,68,69].

Other new biochemical markers that may be informative in the diagnosis and monitoring of cancer patients include vaspin and obestatin. Vaspin is a serine protease inhibitor produced in visceral and subcutaneous adipose tissue [70,71]. To date, only one parallel study has been conducted on vaspin concentrations in saliva and blood in breast cancer patients. Serum vaspin concentrations were shown to be lower in breast cancer patients than in the control group. However, this decrease was not statistically significant. No significant or statistically significant difference in salivary vaspin concentrations was also shown between the breast cancer group and the healthy controls. However, a negative correlation was observed between salivary and serum vaspin concentrations in the control group [9]. The mechanism underlying changes in saliva and blood vaspin concentrations in cancer remains unclear. A number of studies have been conducted on changes in vaspin concentrations in the blood serum of patients with colorectal cancer and medullary thyroid cancer, where vaspin concentrations increased compared to the control group [72]. However, in endometrial cancer, vaspin concentrations were lower compared to the control group [73,74]. Such inconsistent changes in vaspin concentrations in the blood serum, depending on the type of oncology, may be because different biochemical processes occurring in different organs and tissues are involved.

Obestatin is a peptide hormone that controls appetite and is secreted by the salivary glands [75,76]. Thus, serum obestatin concentrations in patients with breast cancer were lower than in the control group, although this decrease was not statistically significant. Salivary obestatin concentrations were higher in patients with breast cancer. It is worth noting that salivary obestatin concentrations were significantly higher than serum obestatin concentrations in both patients with breast cancer and healthy controls, confirming the secretion of obestatin by the salivary glands [9].

## 4. Parallel Studies of Cytokines and Growth Factors in Saliva and Blood in Cancer

There is only one parallel study of the expression of insulin-like growth factor-1 (IGF-1) and insulin-like growth factor binding protein-3 (IGFBP-3) in saliva and serum of patients with oral cancer [77] (Table 2). According to the obtained data, IGF-1 in serum was significantly higher in the healthy control group, and then in descending order, in patients with type 2 diabetes mellitus only (T2DM), T2DM and OSCC, and only OSCC. Moreover, in saliva, the highest value of IGF-1 was among patients with T2DM only, lower content in patients with T2DM and OSCC, and then in descending order: OSCC and healthy controls. Significant trends in changes in IGFBP-3 levels were obtained only in serum, where the highest content was in patients with OSCC and then in descending order: OSCC and T2DM, T2DM, and healthy controls. No significant trends in changes in IGFBP-3 levels were obtained in saliva. Since this is the only study of its kind, it is not possible to draw any conclusions and further research in this area is needed.

Neopterin is another compound that can be used in combination with other indicators for the diagnosis and monitoring of patients with squamous cell carcinoma of the oral cavity and oropharynx [78]. Neopterin belongs to the pteridine compounds. It is considered an activator of T-lymphocytes and macrophages [83]. Its synthesis occurs in human monocytes/macrophages and dendritic cells from GTP (guanosine triphosphate) under the action of the enzyme GTP-CG I (guanosine triphosphate cyclohydrolase I). Induction of the enzyme GTP-CG I occurs under the action of INF-γ, which in turn is activated by T-lymphocytes [84]. In various oncological diseases, neopterin levels increase in serum, saliva, urine and other biological fluids [85,86]. The amount of neopterin is mainly determined by pH and the degree of oxidation [87].

Several studies have reported that IL-6 levels in saliva and serum increase in cancer patients [88] and may also exceed serum IL-6 levels [79]. IL-6 is an inflammatory cytokine. It is produced by lymphoid and non-lymphoid cell types. Primarily, these are T-lymphocytes, macrophages, and fibroblasts, which, under the induction of tumor necrosis factor alpha (TNF-α) and IL-1β, stimulate the production of IL-6 [89]. Activated IL-6 is involved in cell differentiation, proliferation, and apoptosis. It influences the course of chronic inflammation by creating a favorable microenvironment for the tumor, supporting its growth [90]. Increased IL-6 levels have been described in malignant neoplasms of the kidneys, bladder, colon, ovaries, gallbladder, pancreas, lymphatic tissue, and especially in squamous cell carcinoma of the head and neck [91]. IL-6 can be detected in biological fluids such as urine, cerebrospinal fluid, tears, and saliva [92]. The appearance of IL-6 in saliva in cancer patients of various localizations may be due to IL-6 filtrate from serum. Also, oxidative stress increases the amount of ROS, which are transported through the bloodstream to the salivary glands and damage the salivary gland tissue. This, in turn, triggers the recruitment of immune cells, leading to a local inflammatory process in the salivary gland area. Another possible cause may be changes in local immunity and the oral microbiome.

Another promising marker of immune dysfunction in cancer is sHLA-G [81]. Lázaro-Sánchez et al. demonstrated that sHLA-G is elevated in saliva and blood in CRC compared to healthy controls. It was also shown that there is a relationship between the increased concentration of sHLA-G in saliva in CRC and late stages of the disease. In addition, there is a strong correlation between serum and salivary sHLA-G. sHLA-G is a major histocompatibility complex class I molecule. The main role of this molecule is immunosuppression and the formation of tolerance of the immune system. sHLA-G is produced by lymphocytes, macrophages, NK cells, dendritic cells, placental trophoblasts, mesenchymal stem cells, embryonic cells, and tumor cells [93]. It has a low degree of polymorphism and limited expression in tissues, and as a result of alternative splicing of the same mRNA, sHLA-G has 7 isoforms [94,95]. Membrane-associated sHLA-G include G1–G4, while soluble G5–G7 [96]. sHLA-G suppresses allogeneic proliferation of CD4+ T cells [97], inhibits the cytotoxicity of natural killer (NK) and CD8+ T cells [98], and suppresses the maturation of dendritic cells [99] and the activation of B cells [100]. It can also induce apoptosis in antigen-specific CD8+ T lymphocytes [101].

It can be assumed that the high concentration of sHLA-G in saliva in oncological diseases is theoretically due to several mechanisms: direct transport of sHLA-G through the hematosalivary barrier from blood serum or lymph into saliva, destruction of sHLA-G-carrying cells (lymphocytes, macrophages, dendritic cells) in the oral cavity with the release of sHLA-G-loaded vesicles, and penetration of the sHLA-G-carrying vesicles themselves. The specific mechanism of sHLA-G penetration into saliva and other biological fluids remains unclear [102]. The assumption that the main source of sHLA-G in saliva is extracellular vesicles seems the most plausible. It is known that, upon release, vesicles can migrate to recipient cells, overcoming long distances due to the blood or lymph flow [103]. Cancer cells secrete a significant amount of exosomes (extracellular vesicles) compared to healthy cells. These exosomes promote cancer progression by activating anti-apoptotic and oncogenic pathways, namely, invasion, metastasis, and angiogenesis. Exosomes also contribute to immune evasion and are responsible for epithelial–mesenchymal transition [104,105,106,107,108]. Moreover, the increased sHLA-G content in saliva is apparently due to the fact that saliva acts as a filtrate, and therefore, the sHLA-G concentration increases. This makes sense, as a strong direct correlation between the amount of sHLA-G in saliva and blood has been experimentally demonstrated.

Yadav et al. showed that serum and salivary IgA levels are elevated in patients with leukoplakia and squamous cell carcinoma of the oral cavity compared to the control group. However, no correlation was shown between the changes in serum and salivary IgA [80]. Serum IgA is known to be involved in preventing the activation of the complement system, suppressing phagocytosis, chemotaxis, and antibody-dependent cellular cytotoxicity. Thus, the main role of serum IgA is to eliminate antigenic substances without causing an inflammatory reaction [109]. Secretory IgA is produced by mucosal cells to provide local mucosal immunity, which is the first line of defense against pathogens [110]. In the oral cavity, secretory IgA is produced as a dimer by plasma cells localized in the stroma of the salivary glands. Secretory IgA is transported through the secretory epithelium by the polymeric Ig receptor (pIgR) [111]. We suggest that the increase in IgA levels is due to either penetration through the blood-saliva barrier or local secretion in the oral cavity. It is important to note that IgA levels can also be influenced by diet, which affects energy metabolism and immune responses, including IgA production [112]. Thus, an increase in ceramide concentration can induce proliferation of IgA-producing cells [113]. It is also worth considering the degree of clonality and the number of somatic hypermutations in the Ig segment, which has prognostic significance for some types of cancer [114]. Bolotin et al. showed, using TCGA RNA-seq data on melanoma samples, that a high proportion of IgG1 is closely associated with the presence of large clonal expansions. This contributes to a better prognosis. However, a high proportion of IgA correlates with low clonality, which leads to a negative outcome [115]. Thus, high IgA expression and high clonality imply that IgA may be due to a focused immune response and may lead to a better prognosis, whereas high IgA expression and low clonality mean that an immunosuppressive microenvironment stimulates IgA production and probably contributes to unfavorable outcomes in some specific malignancies [116].

Using CRC as an example, a significant increase in salivary and serum chemerin, α-defensin 1, and TNF-alpha was also demonstrated. Waniczek et al. became interested in the macromolecule chemerin due to its biological role [82]. It is capable of activating the G-protein-coupled receptor chemokine-like receptor-1 (CMKLR1) and the G-protein-coupled receptor-1 (GPR1) [117,118,119]. Chemerin, as a chemoattractant, is involved in the formation of innate and acquired immunity. It mainly has a pro-inflammatory effect. TNF-α, IL-1β, IL-6, IL-8, and IFN-γ are regulators of chemerin secretion [120,121,122]. Through the CMKLR1 receptor, chemerin exerts its effects on tissues, leading to stimulation of angiogenesis [123,124].

Defensins are peptides that have pro-inflammatory and cytotoxic effects by stimulating the synthesis of cytokines, chemokines, and growth factors. Defensins also induce the secretion of TNF-α and IFN-γ by neutrophils, which leads to activation and increased phagocytosis in macrophages [125,126]. The group of protein molecules that makes up human neutrophil peptides (HNP) 1–4 is represented by four α-defensin peptides that are expressed in neutrophils, monocytes, lymphocytes, natural killer (NK) cells, and dendritic cells [127]. A number of studies have reported increased expression of α-defensin 1 in several types of cancer, including colorectal cancer [128,129].

TNF-α is the most well-known inflammatory mediator that maintains proper immune system function. Macrophages and tumor cells produce it. TNF-α is associated with all stages of carcinogenesis and is found in many neoplasms [130].

We attribute the increased levels of chemerin, α-defensin 1, and TNF-α in saliva to the small molecular weight of these molecules (chemerin ~16 kDa, α-defensin 1 ~3–4 kDa, and TNF-α ~17 kDa), which facilitates their transport across the blood-saliva barrier [131,132].

## 5. Parallel Studies of Tumor Markers in Saliva and Blood in Cancer

To date, a vast number of studies have examined changes in serum tumor markers. More recently, studies have begun to examine changes in salivary tumor markers. Comparing changes in serum and saliva tumor markers and other parameters is pointless, as these are completely different biological materials and the mechanisms by which certain substances appear are different, although the same pathological process may cause them. Therefore, the directions of change and concentration levels of substances in a given biomaterial may differ. Furthermore, to more fully understand the behavior of specific tumor markers in both saliva and blood during cancer, parallel studies of these parameters in a single patient using these biomaterials are necessary. Research in this area is limited (Table 3).

Laidi et al. evaluated the change in the concentration of CA15-3 in saliva and blood in breast cancer. The concentration of CA15-3 in the serum was statistically significantly higher in the breast cancer group compared to the control group. When measuring the same tumor marker in saliva, no statistically significant differences were found between the groups. Despite this, the scientists showed that there is a strong, direct, statistically significant correlation between serum and salivary CA15-3 [133]. Several independent studies have confirmed the presence of a direct correlation between serum and salivary CA15-3 [9,134]. It was also shown that the most appropriate method for studying and comparing the results of CA15-3 concentration in saliva and blood is to use enzyme-linked immunosorbent assay (ELISA), rather than chemiluminescence assay (CLIA) or electrochemiluminescence assay (ECLIA) [134]. CA 15-3 is an immunogenic region of MUC1. Detection of CA 15-3 in saliva will depend on the extent of damage to the salivary glands and oral epithelium. A greater increase in CA 15-3 in the blood than in saliva may be related to the permeability of the BSB. Its detection may require a more aggressive form of oral inflammation. The condition and number of functioning oral epithelial cells will also influence the concentration of CA 15-3 in saliva.

A study was conducted to determine the concentration of CAE in saliva and serum of patients with breast cancer. Thus, the concentration of CEA in serum was significantly higher in the group of patients with breast cancer and without any significant changes in saliva compared to the control group [9]. CEA levels can be highly dependent on the menopausal status of patients, tumor size, lymph node involvement, and clinical stage. CEA belongs to acidic glycoproteins, which are mainly actively produced during the fetal period. After this period, CEA continues to be produced by cells at low levels, which is considered physiological. An increase in CEA concentration is associated with the presence of benign and malignant diseases [145]. The tumor marker has a high degree of variability at the intermolecular and intramolecular levels [146,147]. Such heterogeneity may be due to different signaling [148]. CEA can be in two forms: membrane-bound (hydrophobic) and soluble (hydrophilic). The transition from the bound form to the soluble form is due to changes in its molecular structure [149]. This transition, depending on whether the cell is normal or cancerous, will have different mechanisms for the formation of the soluble form. One study showed that endogenous glycosylphosphatidylinositol-specific phospholipase D (GPI-PLD) may play a role in the spontaneous release of CEA from human colon cancer cells [150]. Another article reported that phosphatidylinositol-specific phospholipase C plays a key role in the process of CEA release from the cell membrane and its conversion to the hydrophilic form in a human colon adenocarcinoma cell line [151]. While in normal epithelial cells, the hydrophilic form is formed by non-proteolytic cleavage due to the action of endogenous phospholipases [152]. CEA degradation occurs by Kupffer cells (liver macrophages), which possess receptors for CEA, while alveolar macrophages metabolize the tumor marker four times slower [153]. Taking all of the above into account, we can hypothesize both the reasons for the appearance of CEA in saliva and the reasons for changes in its concentration. When a tumor is localized outside the anatomical region of the head and neck, the concentration of CEA in saliva can both increase and decrease. A decrease in the concentration of CEA in saliva may be associated with its degradation by oral macrophages, as well as possible inhibition of the activity of endogenous phospholipases, which are involved in non-proteolytic cleavage. In this case, biochemical changes will occur in the salivary glands and oral epithelium that are not directly related to the oncological process, but indirectly, through increased oxidative stress and the formation of ROS. If the cancer process occurs in the head and neck region, CEA concentrations may be increased due to altered immune responses. Cancerous tumors are known to influence their microenvironment to evade immune defenses through reprogramming. In this case, the macrophages that engulf CEA may be altered such that either MCH II does not recognize CEA or the receptor for this tumor marker is lost. This may also be due to the activation of specific enzymes involved in the formation of the hydrophilic form of CEA.

Vuković et al. showed that in ovarian cancer, the concentration of CA125 in saliva and serum was increased compared to the control group. Moreover, the increase in the level of CA125 in serum correlated with the decrease in the level of CA125 in saliva, although the predictability of the level of CA125 in serum was much higher compared to the level of CA125 in saliva. Specificity was also significantly higher [138]. Other scientists showed that the concentration of CA125 in saliva and serum was significantly increased in patients with untreated breast cancer compared to patients with treated breast cancer and the control group [135]. Conflicting results were obtained by scientists Tay et al. in the study; the levels of CA125 in serum and urine were significantly increased in patients with malignant ovarian cancer compared to patients with benign ovarian tumors. Moreover, no significant differences in salivary CA125 levels were found between these groups [139]. Chen et al., on the contrary, reported that salivary CA125 has a better diagnostic value compared to serum CA125. They observed higher levels of CA125 in saliva and serum in patients with malignant tumors compared to patients with benign tumors. However, no statistically significant correlation was found between serum and salivary CA125 [140]. It is known that CA125 belongs to MUC16 and is the largest membrane-associated mucin in this class. It is expressed on the apical membranes of moist epithelial cells of the human cornea and conjunctiva, on the epithelium of the upper respiratory tract and the epithelium of the female reproductive system [154]. MUC16 is the longest of the membrane-associated mucins [155]. The expression of MUC1 and MUC16 increases in response to infectious damage and oncological processes.

We propose two mechanisms for the increase in CA125 in the blood and saliva depending on the type of malignant neoplasm. For example, in ovarian cancer, cancer cells produce CA125. The tumor process activates the action of TNF-α and IFN-γ. CA125, TNF-α, and IFN-γ enter the bloodstream, where we obtain high values for these indicators. Then, with the blood flow, CA125, TNF-α and IFN-γ reach the BSB and are filtered into saliva. Also, TNF-α and IFN-γ themselves can, through the regulation of transcription mediated by NF-κB, stimulate increased expression of MUC16 in sites unrelated to the tumor process, which will further increase the circulation of CA125 in both the blood and saliva [156]. We also hypothesize that this mechanism will be valid for all tumors localized at the site of MUC16 synthesis. However, if the oncological process occurs in a different location, we will also continue to see an increase in CA125 concentrations in both serum and saliva, but only due to the activation of nonspecific TNF-α and IFN-γ, which will stimulate CA125 expression. It is also possible that CA125 (MUC16) is easier to detect in saliva due to its physiological localization in large quantities near the oral cavity. The large size of mucin also somewhat facilitates the detection of CA125 in enzyme-linked immunosorbent assays.

In this section, estradiol can be considered as a tumor marker due to its functional properties. Estradiol is a biologically active estrogen that promotes cell proliferation and inhibits apoptosis in tumors. Estradiol levels in saliva and blood are higher in patients with breast cancer and metastases [9]. In this case, elevated estradiol levels occur due to increased adipose tissue mass, which is the source of its synthesis. Estradiol concentrations in saliva increase due to its filtration from the blood.

Farahani et al. showed that serum and saliva concentrations of PSA and B2M were significantly higher in prostate cancer patients compared to patients with benign prostatic dysplasia. In both groups, salivary concentrations of PSA and B2M were lower than those in serum, confirming the positive correlation between serum and saliva PSA and B2M [19].

The c-erbB-2 protein belongs to the epidermal growth factor receptor family and is an oncoprotein. This protein is detected in biopsies from women diagnosed with breast cancer [157]. It has been shown that soluble fragments of the oncoprotein can be released from the cell surface and detected in patients with breast cancer. Streckfus et al. detected c-erbB-2 protein in the serum and saliva of patients with breast cancer in significantly higher concentrations than in the control group. The diagnostic value of c-erbB-2 protein was tested by a parallel study on CA 15-3. The study also reported a diagnostic advantage of c-erbB-2 protein over CA 15-3 in identifying patients with carcinoma [136]. Haider et al. showed that patients with breast cancer had high levels of HER-2 and ER in serum and saliva. The results of the study show a direct correlation between serum and salivary ER, serum and salivary HER2, and serum ER and serum and salivary HER2. The study showed that saliva samples could be used to study the immunological parameters of breast cancer [137]. Zanotti et al. in their study demonstrated that patients with oral squamous cell carcinoma (OSCC) have a statistically significant increase in sEGFR in saliva, but not in plasma, compared to the control group [142].

It is known that sEGFR can be present in serum and saliva [158,159]. Its increased expression in tissues is noted during carcinogenesis of the oral mucosa [160,161]. It is known that sEGFR can be released by proteolytic cleavage and cleaved from the surface of tumor cells or by alternative mRNA splicing, thus representing a potential tumor marker in biological fluids [162,163]. There are different isoforms of sEGFR, which have the same molecular mass of 110 kDa, but have different biochemical properties [164]. In this regard, sEGFR can be detected both in healthy individuals (being a “negative biomarker”) and in patients with cancer (being a “positive biomarker”). Some sEGFR isoforms are able to regulate sEGFR activity by modulating EGFR-mediated signaling: sEGFR binding to EGF and TGF-α ligands [165], or sEGFR binding directly to EGFR, which may be attributed to protective functions [166,167]. In this case, sEGFR acts as a “negative biomarker” whose concentration will decrease during the disease. There are also sEGFR isoforms that increase only in oncology and are classified as “positive biomarkers.” Thus, in breast cancer, ovarian cancer, NSCLC, and head and neck cancer, the concentration of sEGFR in the serum is lower compared to healthy controls [168,169,170]. In patients with cervical and gastric cancer, on the contrary, the concentration of sEGFR in the serum is an order of magnitude higher compared to the control group [171,172]. We assume that saliva in this case acts as a “cast,” filtrate, or “sediment” of plasma sEGFR. Moreover, a decrease in the concentration of sEGFR in the blood during oncology is due to the degree of EGFR expression on cells. With a high degree of expression, circulating sEGFR will bind either to ligands or to EGFR on cancer cells. Thus, we will see a relatively “empty” blood test for sEGFR levels, but elevated levels in saliva and tissues. If the cancer process occurs in tissues with low EGFR expression, we assume that the blood sEGFR level will be higher than in the control group, and saliva will contain even higher sEGFR. Unfortunately, to date, there are no parallel studies of saliva and blood sEGFR levels in cancer cells with low EGFR expression to confirm this hypothesis.

A parallel study of the early response gene X-1 (IEX-1) in saliva and blood was conducted in patients with ovarian cancer. The early response gene X-1 (IEX-1) belongs to the early response gene family. IEX-1 is involved in cell growth, differentiation, and apoptosis under conditions of cellular stress. Han et al. showed a significant decrease in IEX-1 expression in epithelial ovarian tumors [141]. This suggests the involvement of IEX-1 in the suppression of epithelial ovarian carcinoma development. These results indicate the clinical potential of IEX-1 expression in blood and saliva as a sensitive and specific diagnostic method for the detection of epithelial ovarian cancer [172].

MiR-21 is a short non-coding RNA molecule. Its functions include gene regulation, cell growth, apoptosis, and the immune response, influencing T-cell differentiation. When overexpressed, it acts as an oncogene. Thus, miR-21 is capable of affecting tumor suppressor genes such as PTEN and PDCD4, inhibiting expression and promoting cell proliferation and survival. Sazanov et al. showed that miR-21 is overexpressed in saliva and plasma in patients with colorectal cancer. It was also noted that the miR-21 expression test in saliva has high sensitivity and specificity compared to the test in plasma [144].

The mean Cyfra 21-1 concentration in serum ranged from 2.81 to 13.33 ng/mL depending on the stage, and in saliva from 14.03 to 35.04 ng/mL, and in both cases, a close correlation with the clinical stage was observed [143]. Receiver-operator curve analysis showed that saliva with Cyfra 21-1 had a higher sensitivity in detecting squamous cell carcinoma compared to serum with Cyfra 21-1 [173]. A significant positive correlation was found between serum and saliva Cyfra 21-1 levels, serum Cyfra 21-1 levels and cytokeratin 19 mRNA expression, and between salivary Cyfra 21-1 levels and cytokeratin 19 mRNA expression. Moreover, the correlation between serum and saliva Cyfra 21-1 levels was highly significant [174]. Cyfra 21-1 is a soluble fragment of cytokeratin 19 that is expressed in all and cancerous epithelial cells. The main functions of cytokeratin 19 are to maintain the integrity of epithelial cells, mediate stress responses, cellular signaling, apoptosis, and participate in the immune response [175,176,177]. The formation of soluble Cyfra 21-1 fragment in malignant epithelial cells occurs through the activation of proteases [178,179]. The appearance of Cyfra 21-1 in saliva when a tumor is located remotely from the salivary glands may be due to the simple filtration of Cyfra 21-1 into saliva due to its elevated blood concentration. It is possible that inflammatory processes, exacerbated by cancer, influence the oral cavity, which damages the salivary gland tissue and oral epithelium, activating immune cells and proteases, leading to an increase in Cyfra 21-1 in saliva. When cancer is localized in the oral cavity, Cyfra 21-1 directly enters the saliva because of destructive processes.

## 6. Conclusions

There are few parallel studies of identical parameters in saliva and blood. These studies were conducted on small and heterogeneous samples, reducing the validity of the results. This review examined changes in enzymes, cytokines, and tumor markers in saliva and blood in the same patients with various oncological diseases. Thus, changes in biochemical parameters in saliva and blood, in some cases, were inconsistent. Moreover, the vast majority of cytokines and tumor markers showed an increased trend in both saliva and blood.

The likely causes of discrepancies in data from different authors may be the heterogeneity of the samples, including the proportion of patients with different disease stages included in the target cohort. Gender, age, and comorbidities, as well as the diagnostic test systems used to measure saliva parameters, are undoubtedly important factors. To ensure comparability of the results of measured parameters in saliva, it is necessary to develop and implement standards for collection (stimulated/unstimulated saliva), storage, transportation and pre-processing of saliva. However, an analysis of the literature suggests that saliva should be considered an independent biological fluid with its own criteria for normal and abnormal results.

## Figures and Tables

**Table 1:** A parallel comparison of biochemical parameters in saliva and plasma in various types of cancer.

Cancer Type	Enzymes and Proteins	Saliva	Blood	Group Characteristics	References
Breast cancer	Alkaline phosphatase	↑	↑	40 females with BC + 20 non-BC females *Age*: No data. *Stage*: St I—3, St II—16, St III—16, St IV—5	Atrooz O. et al., 2025 [7]
	Gamma-glutamyl transferase	↑	↑		
	Aspartate aminotransferase	↑	↑		
	Urea	↑	↑		
	Uric acid	↑	↑		
	Alanine aminotransferase	↓	↓		
	Total protein	↑	↓		
	Albumin	↑	↓		
	Calcium	↓	↑		
	Lactate dehydrogenase	↓	↑		
	Total protein	↓	↑	40 females with BC + 40 non-BC females *Age*: 50–70 years. *Stage*: No data	Al-Muhtaseb S.I. et al., 2014 [8]
	Vaspin	=	↓	30 females with BC + 30 non-BC females. *Age*: 45–70 years. *Stage*: No data	Farahani H. et al., 2020 [9]
	Obestatin	↑	↓		
Gastric and colorectal cancer	Phosphate	↑	↑	Gastric and colorectal cancer—26 (13 men, 13 women) + HC—30 (16 men, 14 women). *Age*: No data *Stage*: No data	Hajali M.H. et al., 2024 [10]
Head and neck cancer	Adenosyl deaminase ADA2	↑	↑	Oral cancer total—41 (36 men, 5 women). *Age*: 47–80 years. *Stage*: No data HC—44 (16 men, 28 women). *Age*: 20–45 years.	Luo W. et al., 2020 [11]
	Magnesium	↓	↓	Precancerous (OSMF + leukoplakia)—17, OSCC—17, HC—17. *Age*: No data. *Stage*: No data	Aziz N.Z. et al., 2018 [12]
	Lactate dehydrogenase	↑	↑	Precancerous (OSMF)—20, HC—20. *Age*: 18–60 years.	Rao K. et al., 2017 [13]
		↑	↑	OSCC—25 (men 10, women 15, mean age 61), HC—25 (men 8, women 17, mean age 42.73). *Stage*: No data	Gholizadeh N. et al., 2020 [14]
	MMP-2	=	=	HNSCC—20, HC—20. *Age*: Mean age 60.95 years. *Stage*: St I—2, St II—6, St III—5, St IV—7	Dalirsani Z. et al., 2019 [15]
	MMP-9	↑	↑		
	MMP-3	↑	↑	Pharynx and larynx cancer—60 (men 55, women 4). *Age*: 32–89 years *Stage*: T: T1—9, T2—25, T3—11, T4—14 N: N0—30, N1—12, N2—13, N3—4 M: M0—59, M1—0 G: G1—18, G2—39, G3—2	Polz-Dacewicz M. et al., 2017 [16]
	MMP-9	=	=		
Glioblastoma	Cyclic-AMP	↑	↑	Glioblastoma—21 (10 men, 11 women). *Age*: 37–82 years *Stage*: No data	Muller Bark J. et al., 2023 [17]
	UDP	↑	↑		
	Cis-aconitate	↑	↑		
	Dihydroorotate	↑	↑		
	3-hydroxy-kynurenine	↑	↑		
	Kynurenine	Higher in blood than in saliva			
	Gycolytic metabolites				
	Creatinine				
	Ceramides	↑	↓		
Non-small cell lung cancer (NSCLC)	Total triglycerides	↑	↑	26 saliva and 25 plasma patient samples	Hwang B.Y. et al., 2023 [18]
	Ceramide species	↓	↓		
	Phosphatidylinositol	↓	↓		
	Phosphatidylserine	=	↓		
Prostate Cancer	Melatonin	↑	↑	Prostate Cancer—20, benign prostatic hyperplasia (BPH)—20 *Age*: 50–55 years *Stage*: No data	Farahani H. et al., 2020 [19]
	Creatine kinase	↑	↑		
	Zinc	↑	↑		
	Creatinine	↑	↑		

**Note.**  For all tables: ↑ increase, ↓ decrease in concentration, = no change in concentration.

**Table 2:** A parallel comparison of cytokines and growth factors in saliva and blood in cancer.

Cancer Type	Cytokines and Growth Factors	Saliva	Blood	Group Characteristics	References
Head and neck cancer	Insulin-like growth factor-1 (IGF-1)	=	↑	HC—12, T2D—12, oral cancer—12, T2D and oral cancer—12 *Stage*: No data	Abhinav R. et al., 2025 [77]
	Neopterin	↑	↑	OSCC—44, oropharyngeal squamous cell carcinoma (OPSCC—6). Males 33, females 17. *Age*: 39–87 years. *Stage*: St I—23, St II—17, St III—3, St IV—6.	Šašková L. et al., 2025 [78]
	Interleukin-6	↑	↑	HC—100 (Males 65, females 35) *Age*: 21–65 years. Leukoplakia—50 (Males 29, females 21) *Age*: 21–90 years. Oral submucous fibrosis (OSMF)—50 (Males 42, females 8) *Age*: 21–70 years. OSCC—100 (Males 68, females 32) *Age*: 21–90 years. *Stage*: St I—32, St II—32, St III—25, St IV—11	Dineshkumar T. et al., 2016 [79]
	sIgA	↑	↑	HC—10, oral leukoplakia—15, OSCC—15 *Stage*: No data.	Yadav P. et al., 2023 [80]
Colorectal cancer (CRC)	sHLA-G	↑	↑	HC—10 Serum: CRC—20 *Stage*: St I+II—8, St III+IV—12 Males 9, females 11 *Age*: <70 years—9, >70 years—11. Saliva: CRC—15 *Stage*: St I+II—5, St III+IV—10 Males 6, females 9 *Age*: <70 years—7, >70 years—8.	Lázaro-Sánchez A. D. et al., 2020 [81]
	Chemerin	↑	↑	CRC—39 Males 18, females 21 *Age:* 67.8 ± 10.3 years. HC—40 Males 19, females 21 *Age*: 64.8 ± 9.4 years. *Stage*: St I—14, St II—9, St III—13, St IV—3.	Waniczek D. et al., 2022 [82]
	α-Defensin 1	↑	↑		
	TNF-α	↑	↑		

**Table 3:** A parallel comparison of tumor markers in saliva and blood in cancer.

Cancer Type	Tumor Markers	Saliva	Blood	Group Characteristics	References
Breast cancer	CA 15-3	=	↑	Breast cancer—29 *Age*: 47.24 ± 9.52 years *Stage*: No data HC—31 *Age*: 43.45 ± 14.72 years	Laidi F. et al., 2014 [133]
		↑	↑	Breast cancer—26 (luminal A-like—7, luminal B-like HER2 negative—3, luminal B-like HER2 positive—4, HER2 positive—5, triple negative—5) *Age*: 48.23 ± 11.51 years *Stages*: St I—2, St IIa—10, St IIb—3, St IIIb—4, St IIIc—1, St IV—5. HC—28 *Age*: 37.64 ± 13.57 years.	Assad D.X. et al., 2020 [134]
		=	=	Breast cancer—30 *Age*: 54.03 ± 8.25 years HC—30 *Age*: 51.30 ± 3.15 years	Farahani H. et al., 2020 [9]
	CEA	=	↑	Breast cancer—30 *Age*: 54.03 ± 8.25 years HC—30 *Age*: 51.30 ± 3.15 years	Farahani H. et al., 2020 [9]
	CA 125	↑	↑	Untreated breast cancer—24 Treated breast cancer—23 HC—25 *Stages*: No data	Agha-Hosseini, F. et al., 2009 [135]
	Estradiol	↑	↑	Breast cancer—30 *Age*: 54.03 ± 8.25 years HC—30 *Age*: 51.30 ± 3.15 years	Farahani H. et al., 2020 [9]
	c-erbB-2	↑	↑	Benign breast lesions—41 Breast cancer—30 HC—57 *Stages*: St 0—1, St I—6, St IIa—8, St IIb—3, St IIIa—2, St IIIb—3, NA—7.	Streckfus C. et al., 2000 [136]
	HER-2	↑	↑	Breast cancer—45 *Age*: 48.27 ± 2.045 years Benign breast lesions—45 *Age*: 36.83 ± 2.583 years HC—40 *Age*: 28.60 ± 1.764 years	Haider M. et al., 2024 [137]
	ER	↑	↑		
	CA 125	↑	↑	Benign breast lesions—48 *Age*: 46.9 ± 16.24 years Breast cancer—50 *Age*: 59.3 ± 14.5 years *Stages*: No data	Vuković A. et al., 2021 [138]
		=	↑	Breast cancer—105	Tay S.K. et al., 1994 [139]
		↑	↑	Benign pelvic masses—92 Malignant pelvic tumors—41	Chen D.X. et al., 1990 [140]
Ovarian tumor	Immediate early response gene X-1 (IEX-1)	↓	↓	Epithelial ovarian carcinoma—56 *Age*: 56 years Benign controls—21 (serous cystadenoma—16, mucinous cystadenoma—5) *Age*: 47 years *Stages*: St I+II—7, StIII+IV—49	Han L. et al., 2011 [141]
Head and neck cancer	sEGFR	↑	↓	OSCC—63 Males 44, females 19 *Age*: 68.6 years HC—60 Males 33, females 27 *Age*: 52.1 years *Stages*: in situ—1, T1—12, T2—12, T3—1	Zanotti L. et al., 2017 [142]
	Cyfra 21-1	↑	↑	OSCC—40 *Age*: <50 years—17, >50 years—23 Males 27, females 13 HC—40 *Age*: <50 years—16, >50 years—24 Males 28, females 12 *Stages*: St I—8, St II—10, St III—9, St IV—5	Adusumilli P. et al., 2023 [143]
CRC	Micro-RNA-21	↑	↑	CRC—31 Males 14, females 17 *Age*: <60 years—6, 61–70 years—14, 71–80 years—7, >81 years—4 HC—34 Males 16, females 18 *Age*: <60 years—11, 61–70 years—13, 71–80 years—6, >81 years—4 *Stages*: St II—6, St III—16, St IV—9	Sazanov A. et al., 2017 [144]
Prostate Cancer	PSA	↑	↑	Prostate Cancer—20, benign prostatic hyperplasia (BPH)—20 *Age*: 50–55 years *Stage*: No data	Farahani H. et al., 2020 [19]
	β-2 microglobulin	↑	↑		

## Data Availability

No new data were created or analyzed in this study. Data sharing is not applicable to this article.
